# The Place for Paediatricians

**Published:** 1992-03

**Authors:** Thomas E. Oppé

**Affiliations:** Emeritus Professor of Paediatrics St. Mary's Hospital Medical School

## Abstract

Professor Victor Neale was a leading paediatrician and respected teacher at a time when the specialty was growing rapidly and employing new professional skills, knowledge and attitudes. The benefits of these developments might be more fully realized by the establishment of a college of Paediatricians. But the consequential loss of affinity with the general medicine of adults and the Royal Colleges of Physicians could weaken the power of the medical profession to maintain the highest standards of medical practice.


### The Place for Paediatricians

### (based on the "Victor Neale Memorial Lecture" given at the meeting of the S.W. Paediatric Club, April 1991)

**Thomas E. Oppé, CBE, FRCP**
Emeritus Professor of Paediatrics
St. Mary's Hospital Medical School

**SUMMARY**
Professor Victor Neale was a leading paediatrician and respected teacher at a time when the specialty was growing rapidly and employing new professional skills, knowledge and attitudes. The benefits of these developments might be more fully realized by the establishment of a college of Paediatricians. But the consequential loss of affinity with the general medicine of adults and the Royal Colleges of Physicians could weaken the power of the medical profession to maintain the highest standards of medical practice.

The Founder of the South West Paediatric Club, Albert Victor NEALE, MD, FRCP was born in Birmingham at the turn of the Century into a large and poorly-off family. As Simon Schama wrote about another distinguished physician "he had not been 'bred' a physician but had made himself into one by hard labour and stubborn determination".[1]

Victor died in 1970, five years after his retirement from the Chair of Child Health at Bristol from which office he had created an Academic Department widely known for innovative teaching and the enthusiastic pursuit of every aspect of child health. In 1960 he was simultaneously President of the British Paediatric Association (BPA), and Dean of the Faculty of Medicine in the University of Bristol.

Following his appointment in 1947 he led the paediatricians in the South-West then few in number and often single-handed. They were assured of his support in their efforts to develop better paediatric services and to retain special clinical or research interests. The S.W. Paediatric Club was a successful means of bringing together paediatricians and doctors from any speciality who shared an interest in the medical care of children and their families.

In retirement he served many important statutory and voluntary bodies and practised his interest in medical history, his love of scholarship and his writing skills. Sadly, one major work; the second volume of the History of the BPA: 1952-1968[2] had to be made ready for publication after his death by his friend and also former BPA President, Dr. Alfred White Franklin.

Victor Neale was justifiably proud of his place as a distinguished member of an esteemed profession. His life and work fully implemented the ethos of the medical profession and of children's doctors in particular. His loyalty to the professional and academic institutions to which he belonged was absolute but he disliked empty ceremonies and rituals based on arrogance and false traditional values.

### PAEDIATRICS IN THE NHS

Children and their doctors got great benefits from the advent of the National Health Service (NHS). The 1946 Act made it the duty of the Minister of Health, "to promote the establishment in England and Wales of a comprehensive health service . . . and for that purpose to provide or secure the effective provision of services".[3]

At one stroke children had access 'free of charge' to a range of paediatric care that improved rapidly under the influence of NHS consultant paediatricians who now had parity of esteem and conditions of service with consultants in other acute specialties.

Most leading paediatricians (including Victor Neale) in the 1950's-1970's believed that paediatrics with its well regarded professional body (the BPA) was satisfactorily situated within the organisation of the medical profession to ensure progress.

### PAEDIATRIC ASPIRATIONS

Indeed many long-standing aspirations of the British Paediatric Association (founded 1928) gradually became realised. The General Medical Council (G.M.C.) endorsed Paediatrics (Child Health) as one of the five major undergraduate clinical disciplines with a mandatory if imprecise place in the Final examination.

The establishment in every Medical School of an Academic Department of Paediatrics and/or Child Health was deemed essential to the sufficient performance of these educational functions: some of the London Medical Schools being the last to fulfil this requirement.

In matters of post-graduate training and specialist accreditation paediatrics was until recently managed as a branch of general medicine akin to system specialties such as dermatology and endocrinology. As the distinction between the medicine of childhood and that of adults became progessively better understood a greater measure of professional independence was achieved.

A paediatric option for the MRCP (UK) Part 2 examination was obtained and within its general format paediatricians now run this option which is passed by about 225 candidates each year.

Inspection and approval of training posts in paediatrics, and advice to Advisory Appointment Committees about NHS paediatric posts are now predominantly in paediatric hands, with the Royal College of Physician's Regional Paediatric Advisers playing a major part.

Paediatricians as individuals and collectively through the BPA became increasingly involved in advising government and other official bodies on matters of child health but had no formal recognition in the professional establishment.

### "THE PROFESSION"

The 1946 Act obliged the Minister to seek the advice of 'The Profession' on the discharge of his duties, and to keep developments under review. The statutory Bodies set up for this purpose were The Central Health Services Council and its professional Standing Advisory Committees.[4] The sources of professional power considered by Government as 'The Profession' are; the Medical Colleges and their Faculties, the General Medical Council (GMC), and the British Medical Association (BMA).

Professional standards are a main concern of the Colleges and Faculties: the Royal College of Physicians of London (RCP Lond) of which the majority of consultant paediatricians are Fellows or Members declares that it "sets the standards and controls the quality of medical practice in hospitals in England, Wales and Northern Ireland. It conducts examinations, training, education and research in medicine. It advises the Government, the public and the profession on health and medical matters".[5] A Paediatric Board, Paediatric Regional Advisers, the regular appointment of a Paediatric Censor and now a Paediatric Vice-President exert considerable Paediatric influence on the business of the RCP (London). The special interests of Paediatrics are served somewhat differently in the Scottish Colleges (Edinburgh and Glasgow), notably by the recent establishment of the Joint Paediatric Committee of the Scottish Royal Colleges (JPCSRC).

The GMC a statutory body created as the General Council of Medical Education and Registration to implement the Medical Act of 1855 is now much occupied with the offspring, the Medical Act of 1983.

The GMC enshrines the principle that "independent, self-regulation is the best system . . . . because it promotes the highest possible professional standards".[6] Paediatrics is not formally represented on the GMC: elected or nominated members who happen to have an interest in or experience of paediatric practice may contribute to the Council's deliberation of paediatric matters. Currently there are three paediatricians and one paediatric surgeon among the 100 or so members.[6]

The BMA was set up in 1832 and is "the recognised spokesman for all doctors . . . . to the public at large, the Government, employers, MPs and the media". As such its role in 'The Profession' is seen largely as the maintenance of "the honour and interests of the medical profession".[7] Children's doctors have no special representation in the complex committee structure although paediatric matters are frequently on the scientific and the political agenda.

### PROFESSIONAL RIGHTS AND DUTIES

The late Paul Sieghart defined a profession as "composed of people who are experts in a discipline that confers powers to do both good and harm, who practice that discipline for the benefit of others, who choose to give the interests of those others consistent precedence over their own and who seek to limit the harm they might do by submitting to a set of ethical rules designed to serve the paramount interest of some noble cause".[8] These values are so recognised in our Society that the professions to a substantial degree are enabled to determine their own conditions for entry, and to establish codes of professional practice enforceable through self-regulated tribunals.[9]

Doctor power and privilege are not universally acclaimed as evidenced by Professor Titmuss' references to the "medical priesthood": his observation that "Increasing division of labour, mainly (although not always) based on a scientific rationale . . . . has resulted in great benefits in the reduction and alleviation of suffering and disease. Science and specialisation have had . . . . a profound influence not only on the practice of medicine and to the extent to which its objectives are realizable, but on the established patterns of relationships and behaviour within the profession itself" was prophetic.[10]

One effect of increasing specialisation has been the inability of the long established Royal Colleges — the RCP (London), founded 1518; and the RCS (England), founded in 1745, to contain the professional aspirations of; anaesthetists, obstetricians and gynaecologists, pathologists, psychiatrists and of radiologists. Each of these groups has formed a College of its own, as did general practitioners: public health doctors and ophthalmologists set up Faculties within a parent College.

### A COLLEGE FOR PAEDIATRICIANS?

The notion that there should be a College (or a Faculty) of Paediatrics has been mooted intermittently since 1943, but it was not until 1977 that the opinion of the membership was formally sought by the Council.[11] Much inconclusive and divisive discussion followed from which emerged in 1991 a draft prospectus for Collegiate status. If the membership approves the BPA Council will be empowered to proceed toward the foundation of yet another medical College. Is this only a ploy to increase the 'clout' of paediatrics in the corridors of power or is there also a convincing moral justification for the endeavour?

I shall try to answer this question by returning to Sieghart's definition. Formation of a new College would be morally desirable only if paediatricians served a particular "noble cause", and also possessed special expertise without which the paramount interests of the beneficiaries would be imperfectly attained. In essence then is a College of Paediatricians needed to confer on the practice of the medicine of childhood sufficient powers "to do good" and "to limit the harm".

### The Noble Cause

I suspect that Victor Neale would have whole-heartedly endorsed Article 24 of the United Nations Convention on the Rights of the Child (1990) which recognises "the right of the child to the enjoyment of the highest attainable standard of health and to facilities for the treatment of illness and rehabilitation of health". For paediatrics to devote its expertise in pursuit of this mission is surely a sufficiently noble cause?

### The Expertise

Paediatricians possess the special knowledge, skills, and attitudes needed for many aspects of the health care of children and they form the professional body most competent to advise on such matters as the allocation of resources to the delivery of paediatric care, and the difficult ethical problems associated with the welfare of children.

These seem good enough reasons for a College that should work with, but be independent of other Medical Colleges. Nevertheless I fear that a proliferation of Colleges each with a somewhat different mission will diminish rather than enhance the beneficial influence of professionally driven standards on a medical practice that is increasingly politicised and commercialised. There must also be some doubt as to the ability or the willingness of the 1708 Ordinary Members of the BPA (of whom about 1,000[12, 13] are consultant paediatricians) to underwrite the costs of establishing and running a new College.

I hope it may not be too late for the Royal Colleges to make further constitutional changes so as to encompass fully the professional interests of both adult and child physicians.

### ENVOI

Victor Neale earlier than many of his contemporaries foresaw the enormous expansion in paediatric science and practice, the increasing importance of prevention and the need for specialised paediatric participation in the community. Although robustly child-centred he never neglected to consider the welfare of the whole family, and the influence of the environment.

His conviction that clinical academics are the cream of the medical profession and that paediatricians are the best of physicians was endearingly elitist. I am certain that he would not have supported a change that would separate paediatricians from fellow physicians, and might disrupt the ability of the medical profession to look after the continuities of health and disease that take our patients through childhood to old age.

### REFERENCES

1. SCHAMA S. Death of a Harvard man. *In: Granta 34.* London: Granta Publications, 1991; 14-76.
2. NEALE, A. V. The British Paediatric Association 1952-1968; London; Pitman 1970.
3. Great Britain, Parliament. The National Health Service Act 1946; (9&10 Geo. 6, Ch. 81 Pt.1). London: HMSO 1946.
4. LEVITT R. The reorganised National Health Service; London: Croom Helm 1976.
5. Royal College of Physicians of London. Annual report 1990: London RCP (Lond) 1990.
6. KILPATRICK, R. President's Foreword. *In: General Medical Council Annual Report 1990;* London: GMC 1990.
7. British Medical Association. Membership benefits 1990-1991; London: *BMA* 1990.
8. SIEGHART, P. Professional ethics — for whose benefit? *Journal of Medical Ethics:* 1982, **8:** 25-32.
9. TITMUSS, R. M. The National Health Service in England. *In: Essays on the welfare state 2nd edition;* London: Unwin 1963.
10. FORFAR, J. O.; JACKSON, A. D. M.; LAURANCE B. M. The British Paediatric Association 1928-1988. London BPA 1989.
11. Department of Health. Medical & Dental staffing prospects in the NHS in England & Wales 1989. *Health Trends:* 1990; **22;** 96-103.
12. British Paediatric Association. Paediatric medical staffing for the '90's. London: BPA 1991.

